# Performance Criteria for the Identification of Inertial Sensor Error Models

**DOI:** 10.3390/s19091997

**Published:** 2019-04-29

**Authors:** Oleg Stepanov, Andrei Motorin

**Affiliations:** CSRI Elektropribor, JSC, ITMO University, 190000 Saint Petersburg, Russia

**Keywords:** inertial sensor, filtering algorithm, sensor error model, identification, Bayesian approach, performance criteria

## Abstract

This paper considers performance criteria for the identification of sensor error models and the procedure for their calculation. These criteria are used to investigate the efficiency of the identification problem solution, depending on the initial data, and to carry out a comparative analysis of various suboptimal algorithms. The calculation procedure is based on an algorithm that solves the joint problem of hypothesis recognition and parameter estimation within the Bayesian approach. A performance analysis of the models traditionally used to describe errors of inertial sensors is given to illustrate the application of the procedure for the calculation of performance criteria.

## 1. Introduction

Currently, algorithms based on system description in state space are widely used in control and estimation problems. To estimate the state vector of such systems, linear and nonlinear stochastic Bayesian filtering algorithms, such as the Kalman filter, unscented Kalman filter, and particle filter [1,2,3,4], which require the derivation of system models in state space in the form of random processes, are often used. For example, such a problem arises during the processing of redundant measurements, in particular, navigation data fusion, which involves signals from multiple sensors [5,6,7]. To design such stochastic filtering algorithms, we need to describe sensor errors. This, in turn, generates the need to solve the identification problem of their models. The problem aims to reveal the components of sensor errors, i.e., to determine the model structure and estimate the unknown parameters of its components based on the data obtained during the tests. The data are usually obtained by comparing the readings of the sensors with reference values, and as a result only contain information about sensor errors. Common tests, possible structures of error models, and the corresponding parameters to be identified, as well as some methods for their derivation, are given, for example, in [8,9]. The review shows that methods based on the analysis of sample characteristics are widely used in applied problems concerned with the processing of measurement data. Such sample characteristics include power spectral density [10,11], Allan variance (AV) [12,13,14,15,16,17,18], etc. These methods are usually effective only for stationary ergodic processes; in addition, they require long-term measurements. Moreover, in some cases the obtained model cannot be applied directly to Bayesian stochastic filtering algorithms [1,2,3,4,5,6,7,19,20] that imply the description of errors in time domain using the shaping filter defined in state space. The wavelet analysis is becoming increasingly popular nowadays [21]. The approach proposed in [21,22,23] delivers a global selection criterion based on the wavelet variance that can be used to design an algorithm for automatic identification of a model structure.

Many methods for the identification of state space systems have been developed so far [24,25,26,27,28,29]. For example, [1,30,31,32] formulate the problem of state space model identification within the framework of the Bayesian approach as a joint problem of hypotheses about the model structure recognition and estimation of its parameters. The corresponding problem statement as well as its solution algorithms are inherently close to the statements and algorithms considered in tracking problems [33,34,35,36] or system diagnostics [37,38]. 

However, such methods have not been properly applied for the identification of sensor error models. In [39] and [40] the authors use the Bayesian approach to estimate sensor error parameters only. In [41,42] the authors show that the problem can be generalized for the identification of the sensor error model structure as a joint problem of hypothesis recognition and parameter estimation. The problem solution within the state space and Bayesian formulation allows us to calculate directly the probabilities of correct structure identification in order to choose the sensor error model. The obtained estimates of the model parameters are the optimal Bayesian estimates for any time interval. The corresponding root-mean-square (RMS) of estimation errors can also be calculated both for stationary and nonstationary models in the frames of this approach.

In this paper we propose a method for determining the estimation quality for the problem of sensor error model identification. The straightforward calculation of the structure identification probabilities allows us to compute the unconditional probability for correct model identification and unconditional relative standard deviations for the estimated parameters. These unconditional characteristics can be used as identification quality criteria. The criteria can be computed by simulation to answer the question of whether it is possible in principle to determine the model and its parameters under certain conditions. Thus, they provide a quantitative measure of possibility to identify a model under certain conditions and evaluate the quality of such problem solution within a certain suboptimal algorithm such as the AV.

The Bayesian approach-based algorithm for identification of a sensor error model was considered in [42], but in this paper we focus on obtaining performance criteria for solution of the identification problem, propose methods for their calculation and give an example of their practical application.

The paper is structured as follows: Part 2 considers the statement of the identification problem for the sensor error model and estimation of its parameters in the framework of the Bayesian approach. The general algorithm for its solution is described here as well. Part 3 introduces the quality criteria for the problem and the method for their calculation. Part 4 illustrates the proposed method by the example of the identification problem solution for models traditionally used to describe the errors of navigation sensors.

## 2. Problem Statement and Solution Algorithm

To state the problem within the Bayesian approach, let us introduce the following model. Assume that we have a set of hypotheses about sensor error models, one of which is true. Moreover, each model can be represented in state space by the shaping filter [39,41]:

(1) $$\begin{array}{l} x_{i}^{k}=\Phi_{i}^{k}(\theta^{k})x_{i-1}^{k}+\Gamma_{i}^{k}(\theta^{k})w_{i}^{k},\\ \theta_{i}^{k}=\theta_{i-1}^{k}=\theta_{}^{k}, \end{array}\}$$

(2) $$y_{i}=H_{i}^{k}(\theta^{k})x_{i}^{k}+\Psi_{i}^{k}(\theta^{k})v_{i}^{k}$$

where $y_{i}^{}$ is a sensor error sample obtained in the tests; $x_{i}^{k}$ is the error model state vector; k is the number of the hypothesis that specifies the structure of the sensor error model, $k=\bar{1\ldots K}$ is a natural number from 1 to *K*; $\Phi_{i}^{k}(\theta^{k})$, $\Gamma_{i}^{k}(\theta^{k})$, $H_{i}^{k}(\theta^{k})$, $\Psi_{i}^{k}(\theta^{k})$ are the matrices describing the shaping filter for this model, which are nonlinearly dependent on the constant vector of parameters $\theta^{k}$ and time instance *i*; $w_{i}^{k}$ is $p_{}^{k}$-dimensional system noise; $v_{i}^{k}$ describes the $m_{}^{k}$-dimensional white-noise component of the sensor error. Both are discrete zero-mean Gaussian white noise with identity covariance matrices. Vector $x_{i}^{k}$ is assumed to be Gaussian with known prior expectation and covariance. Prior probability density function (PDF) for $\theta^{k}$ is also known. Note that for different hypotheses the dimensionality of vectors $\theta^{k}$ and $x_{i}^{k}$ can also be different and the model can be time-varying because matrices $\Phi_{i}^{k}(\theta^{k})$, $\Gamma_{i}^{k}(\theta^{k})$, $H_{i}^{k}(\theta^{k})$, $\Psi_{i}^{k}(\theta^{k})$ can depend directly on time instant *i*.

Under the assumptions made, the problem of model identification can be formulated as a joint problem of hypotheses recognition and parameter estimation, i.e., the problem of determining the hypothesis number *k*, which is best suited for measurements $Y_{i}={\left[\begin{array}{ccc} y_{1} & \ldots & y_{i} \end{array}\right]}^{T}$, and estimating the corresponding vectors $\theta^{k}$ and $x_{i}^{k}$. Thus, we can completely describe the sensor error model if we know the hypothesis number and the corresponding vector $\theta^{k}$ estimate.

Such a problem can be solved with the algorithm described in [40,42]. It allows us to obtain the number of hypothesis that corresponds to the maximum posterior probability and the optimal Bayesian estimates corresponding to the hypothesis vectors ${\hat{\theta }}^{k}$ and $x_{i}^{k}$. The algorithm starts with definition of initial conditions, which is followed by sequential calculation of the hypotheses probabilities and estimates of parameter and state space vectors for each time.

First, a set of *K* hypothesis models Equations (1) and (2) is defined, which is potentially suitable as a sensor error model. Prior PDFs for the vectors $\theta^{k}$ and $x_{0}^{k}$ are usually defined as uniform and Gaussian ones. Their parameters can be roughly estimated using sample characteristics. The PDFs for vectors ${\hat{\theta }}^{k}$ are approximated using, for example, the point-mass method [1,2,43]:

(3) $$f_{\theta^{k}}\left(\theta_{}^{k}/Y_{i},H=h_{k}\right)\approx \sum_{j=1}^{M_{k}}\mu_{i}^{kj}\delta \left(\theta^{k}-\theta^{kj}\right)$$

where $\theta^{kj}$, $j=\bar{1\ldots M_{k}}$ is a grid for $\theta^{k}$ derived on a domain of its PDF within a fixed hypothesis $h_{k}^{}$, $\mu_{i}^{kj}$ are PDF approximation weights. The initial weights $\mu_{0}^{kj}$ corresponding to the prior PDF are defined as equal and normed ones, so that $\sum_{j=1}^{M_{k}}\mu_{i}^{kj}=1,\;\forall \;k$. The Monte Carlo method can also be used for such approximation [1,44,45]. The feature of the algorithm is the subsequent sequential computation of the posterior PDF approximations weights $\mu_{i}^{kj}$ for vector $\theta^{k}$ with $k=\bar{1.K}$, which yields the required estimates and hypotheses probabilities. It should be noted that for a fixed value $\theta^{kj}$, the problem of vector $x_{i}^{k}$ estimation in Equations (1) and (2) becomes linear and can be solved using a Kalman filter (KF). This method is also referred to as the Rao-Blackwellization procedure [1,44]. Furthermore, we describe this recursive procedure, which consists of five steps.

In the first step, matrices $\Phi_{i}^{k}(\theta^{kj})$, $\Gamma_{i}^{k}(\theta^{kj})$, $H_{i}^{k}(\theta^{kj})$, $\Psi_{i}^{k}(\theta^{kj})$ are calculated for the whole set of values $\theta^{kj}$ within the set of grids of all hypotheses. For simplicity, hereinafter, these matrices are referred to as $\Phi_{i}^{kj}$, $\Gamma_{i}^{kj}$, $H_{i}^{kj}$, $\Psi_{i}^{kj}$. Thus, the KF bank is formed in accordance with shaping filters described by Equations (1) and (2), and the estimates of vectors $x_{i}^{kj}$ and their covariances $P_{i}^{kj}$ are calculated using KF equations:

(4) $$\begin{array}{l} {\hat{x}}_{i/i-1}^{kj}=\Phi_{i}^{kj}{\hat{x}}_{i-1}^{kj},\\ {\hat{x}}_{i}^{kj}={\hat{x}}_{i/i-1}^{kj}+K_{i}^{kj}(y_{i}-H_{i}^{kj}{\hat{x}}_{i/i-1}^{kj}), \end{array}$$

(5) $$K_{i}^{kj}=P_{i/i-1}^{kj}{\left(H_{i}^{kj}\right)}^{T}{\left(H_{i}^{kj}P_{i/i-1}^{kj}{\left(H_{i}^{kj}\right)}^{T}+\Psi^{kj}{\left(\Psi^{kj}\right)}^{T}\right)}^{-1}$$

(6) $$\begin{array}{l} P_{i}^{kj}=(E-K_{i}^{kj}H_{i}^{kj})P_{i/i-1}^{kj},\\ P_{i/i-1}^{kj}=\Phi_{i}^{kj}P_{i-1}^{kj}{\left(\Phi_{i}^{kj}\right)}^{T}+\Gamma_{i}^{kj}Q_{i}^{k}{\left(\Gamma_{i}^{kj}\right)}^{T}, \end{array}$$

Here, the upper indexes are used to denote that Equations (4)–(6) correspond to the linear filtering problem within the fixed grid values of vector $\theta^{kj}$ and hypothesis $h_{k}^{}$. The innovations

(7) $$\lambda_{i}^{kj}=y_{i}-H_{i}^{kj}{\hat{x}}_{i/i-1}^{kj}$$

and the corresponding covariances

(8) $$\Lambda_{i}^{kj}=H_{i}^{kj}P_{i/i-1}^{kj}{\left(H_{i}^{kj}\right)}^{T}+\Psi_{i}^{kj}R_{i}^{kj}{\left(\Psi_{i}^{kj}\right)}^{T}$$

are also computed in this step using estimates ${\hat{x}}_{i}^{kj}$ and the corresponding covariances $P_{i}^{kj}$.

In the second step, the values of likelihood functions

(9) $$\begin{array}{l} f_{y_{i}}(y_{i}/Y_{i-1},H=h_{k},\theta_{}^{kj})=N\left(\lambda_{i}^{kj};0,\Lambda_{i}^{kj}\right),\\ f_{x_{i}^{k}}\left(x_{i}^{k}/Y_{i},H=h_{k},\theta_{}^{kj}\right)=N\left(x_{i}^{k};{\hat{x}}_{i}^{kj},P_{i}^{kj}\right) \end{array}$$

are calculated for the whole set of values $\theta^{kj}$ by using estimates ${\hat{x}}_{i}^{kj},$ innovations $\lambda_{i}^{kj}$, and the corresponding covariances $P_{i}^{kj}$, $\Lambda_{i}^{kj}$. Here notation $N\left(x;\bar{x},P\right)$ means the Gaussian function of random vector x with expectation $\bar{x}$ and covariance matrix P. The PDFs in Equation (9) are Gaussian relative to $\lambda_{i}^{kj}$ and $x_{i}^{k}$, respectively, because the problem considered in Equations (1) and (2) is linear for fixed values of $\theta^{kj}$.

In the third step, the posterior probabilities of all hypotheses $h_{k}^{}$ are calculated according to

(10) $$\mathrm{Pr}(H=h_{k}/Y_{i})\approx \frac{\left[\sum_{j=1}^{M_{k}}\mu_{i-1}^{kj}N\left(\nu_{i}^{kj};0,\Lambda_{i}^{kj}\right)\right]\mathrm{Pr}(H=h_{k}/Y_{i-1})}{\sum_{k=1}^{K}\left[\left[\sum_{j=1}^{M_{k}}\mu_{i-1}^{kj}N\left(\nu_{i}^{kj};0,\Lambda_{i}^{kj}\right)\right]\mathrm{Pr}(H=h_{k}/Y_{i-1})\right]}$$

using the values of the likelihood functions (Equation (9)) computed in the previous step. Equation (10) can be obtained from Bayes’ rule [1,2], taking into account that the likelihood function for each hypothesis can be calculated as:

(11) $$f_{y_{i}}\left(y_{i}/Y_{i-1},H=h_{k}\right)\approx \sum_{j=1}^{M_{k}}\mu_{i-1}^{kj}N\left(\nu_{i}^{kj};0,\Lambda_{i}^{kj}\right)$$

At the same step, hypothesis $h_{k}^{*}$ with the highest computed probability $\mathrm{Pr}(H=h_{k}^{*}/Y_{i})$ is detected. However, the decision that the model corresponding to the hypothesis is true, is only made when $\mathrm{Pr}(H=h_{k}^{*}/Y_{i})\geq \gamma$, where γ is a threshold close to unity. As a rule, the value of γ is assumed to be about 0.8.

In the fourth step, the approximation coefficients $\mu_{i}^{kj}$ for posterior PDFs of vectors $\theta^{k}$ are computed according to Bayes’ rule:

(12) $$\mu_{i}^{kj}=\frac{\mu_{i-1}^{kj}f_{y_{i}}\left(y_{i}/Y_{i-1},H=h_{k},\theta^{kj}\right)}{\sum_{j=1}^{L}\mu_{i-1}^{kj}f_{y_{i}}\left(y_{i}/Y_{i-1},H=h_{k},\theta^{kj}\right)}.$$

In the fifth step, the estimates of vectors $\theta^{k}$, $x_{i}^{k}$ and their covariances are calculated according to the following equations:

(13) $${\hat{\theta }}_{i}^{k}(Y_{i})=\sum_{j=1}^{M_{k}}\mu_{i}^{kj}\theta_{}^{kj},\;{\hat{x}}_{i}^{k}(Y_{i})=\sum_{j=1}^{M_{k}}\mu_{i}^{kj}{\hat{x}}_{i}^{kj},$$

(14) $$\begin{array}{l} P_{i}^{\theta k}(Y_{i})=\sum_{j=1}^{M_{k}}\mu_{i}^{kj}\theta_{}^{kj}{\left(\theta_{}^{kj}\right)}^{T}-{\hat{\theta }}_{i}^{k}{\hat{\theta }}_{i}^{k}{}^{T},\\ P_{i}^{xk}(Y_{i})=\sum_{j=1}^{M_{k}}\left[\mu_{i}^{kj}\left({\hat{x}}_{i}^{kj}{\left({\hat{x}}_{i}^{kj}\right)}^{T}+P_{i}^{xkj}\right)\right]-{\hat{x}}_{i}^{k}{\left({\hat{x}}_{i}^{k}\right)}^{T} \end{array}$$

where the approximation coefficients $\mu_{i}^{kj}$ obtained in the previous step are used. 

As follows from the presented expressions, the algorithm has a high computational complexity. In order to overcome this drawback, in future works it is supposed to design special procedures aimed at reducing the amount of computations.

## 3. Performance Criteria Calculation

The performance criteria for identification of stochastic model described by Equations (1) and (2) can be determined separately: firstly, for the probability of the true model structure identification, and secondly, for the accuracy of the model parameter estimates. For the model structure, the unconditional probability of the identification error can be used as a performance criterion. This probability can be computed directly using the method of statistical trials:

(15) $$1-{\bar{{\mathrm{Pr}}_{i}}}^{R}\left(H=h_{k}\right)\approx 1-\frac{L^{*}}{L},$$

where $L^{*}$ is the number of samples by which the algorithm identified the models correctly, and *L* is the total number of the simulated samples. The accuracy of the model parameter estimates can be evaluated using the unconditional covariance matrix of estimation errors:

(16) $$G_{i}^{\theta k}=E\left\{(\theta^{k}-{\hat{\theta }}_{i}^{k}){\left(\theta^{k}-{\hat{\theta }}_{i}^{k}\right)}^{T}\right\},$$

where E{•} is an expectation sign. Thus, the diagonal elements of this matrix will be used for parameter estimates as a performance criterion. The elements can also be computed using the method of statistical trials:

(17) $$G_{i}^{\theta k}=G_{i}^{R\theta k}\approx \frac{1}{L-1}\sum_{l=1}^{L}\left(\theta^{k}(l)-{\hat{\theta }}_{i}^{k}(Y_{i}^{l})\right){\left(\theta^{k}(l)-{\hat{\theta }}_{i}^{k}(Y_{i}^{l})\right)}^{T}$$

where $\theta^{k}(l)$ is a true value of the unknown parameters vector in sample *l*, ${\hat{\theta }}^{k}(Y_{i}^{l})$ is the estimate of vector $\theta^{k}(l)$ computed using an algorithm and measurements $Y_{i}^{l}$. These elements determine the variance of the estimation errors for the components of the unknown parameters vector. In what follows, we call the performance criteria obtained using Equations (15) and (17) a real identification error probability and a real estimation error variance, correspondingly; they will be indexed with “*R*”.

The unconditional performance criteria in Equations (15) and (16) can also be computed using the probability (Equation (10)) and covariances (Equation (14)) from the above algorithm and the method of statistical trials:

(18) $$1-{\bar{{\mathrm{Pr}}_{i}}}^{C}\left(H=h_{k}\right)\approx \frac{1}{L}\sum_{l=1}^{L}\left(1-{\mathrm{Pr}}_{i}^{l}(H=h_{k}^{*}/Y_{i}^{l})\right),$$

(19) $$G_{i}^{\theta k}=G_{i}^{C\theta k}=\int P_{i}^{\theta k}(Y_{i})f(Y_{i})dY_{i}\approx \frac{1}{L}\sum_{l=1}^{L}P_{i}^{\theta k}(Y_{i}^{l})$$

where ${\mathrm{Pr}}_{i}^{l}(H=h_{k}^{*}/Y_{i}^{l})$ is the probability of the true hypothesis $h_{k}^{*}.$ From here on, the values of the performance criteria obtained from Equations (15) and (19) will be called the calculated identification error probability and the calculated estimation error variance, correspondingly, and they will be indexed with “*C*”. The coincidence of the real and calculated performance criteria is evidence in favor of the validity of the results obtained.

For a particular performance analysis, when the mean value of the unknown parameter is not zero, it is useful to compare not variances, but the relative standard deviation (RSD), which is defined as the ratio of the standard deviation to the mean [46]:

(20) $$V_{i}^{\theta k}(j)=\sqrt{G_{i}^{\theta k}(j,j)}/E\{\theta_{}^{k}(j)\}$$

where *j* is the number of vector $\theta^{k}$ component, $G_{i}^{\theta k}(j,j)$ is the element of Equation (16) with index (j,j). Note that depending on the procedure by which Equation (16) was calculated, we will differentiate between the real RSD, corresponding to the real covariance matrix (Equation (17)), and the calculated RSD, corresponding to the calculated matrix (Equation (19)).

## 4. Examples of Performance Criteria Use

To illustrate the use of the performance criteria, we consider two hypotheses corresponding to the shaping filters:

(21) $$\left\{ \begin{array}{l} x_{i1}^{1}=x_{(i-1)1}^{1}+\sigma_{w}^{}w_{i}^{1},\\ x_{i2}^{1}=x_{(i-1)2}^{1}, \end{array}\right.$$

(22) $$\left\{ \begin{array}{l} x_{i1}^{2}=\exp(-\Delta t/\tau_{m})x_{(i-1)1}^{2}+\sigma_{m}^{}\sqrt{1-\exp(-2\Delta t/\tau_{m})}w_{i}^{2},\\ x_{i2}^{2}=x_{(i-1)2}^{2}, \end{array}\right.$$

and measurements, which represent a sample of a total sensor error, in the form:

(23) $$y_{i}^{}=x_{i1}^{k}+x_{i2}^{k}+\sigma_{v}^{k}v_{i}^{k},\;k=1,2.$$

In the above relations, $w_{i}^{k}$, $v_{i}^{k}$ are independent of each other zero-mean Gaussian white noises with unit variances; $\sigma_{w}^{}$ is the RMS deviation of the system noise for the random walk; τ, $\sigma_{m}^{}$ are the correlation interval and the RMS deviation of the first-order Markovian process in Equation (22), respectively; $\sigma_{v}^{k}$ is the RMS deviation of the white-noise component, Δt is the discretization interval. These models are quite common to navigation sensor error models that usually contain constant bias, drift, and white-noise components [8,12].

The identification problem for this case can be formulated as follows. Find the number of the hypothesis corresponding to the maximum posterior probability $\mathrm{Pr}(H=h_{k}/Y_{i})$, optimal Bayesian estimates for the state vector $x_{i}^{k}={\left[\begin{array}{cc} x_{i1}^{k} & x_{i2}^{k} \end{array}\right]}^{T}$ and the vector of parameters $\theta^{k}$ for this hypothesis using the given measurements $Y_{i}={\left[\begin{array}{ccc} y_{1} & \ldots & y_{i} \end{array}\right]}^{T}$. Thus, each hypothesis corresponds to the different sensor error models. The proposed algorithm chooses one of them using the posterior probability $\mathrm{Pr}(H=h_{k}/Y_{i})$ and estimate the corresponding parameters ${\hat{\theta }}^{k}$. However, the aim of the simulation is to calculate the performance criteria (Equation (15)), and Equations (17)–(20) to show the possibility of identifying sensor error models in certain conditions.

To show how the estimation accuracy changes with the changes in the parameter values, we consider three different cases. In the first case, the RMS of the white-noise component is assumed to be zero $\left(\sigma_{v}^{k}=0,\;k=1,2\right)$. Note that we used the KF Equation (Equation (5)), in which the noise covariance matrix is not inverted. The vectors of unknown parameters $\theta^{k}$ are defined as $\theta^{1}=\sigma_{w}$ for model (22) and as $\theta^{2}={\left[\begin{array}{cc} \tau_{m} & \sigma_{m} \end{array}\right]}^{T}$ for model (22). Both vectors are uniformly distributed with elements within the ranges of $\sigma_{w}\in \left[\begin{array}{cc} 0.04 & 0.15 \end{array}\right]$ u, $\tau_{m}\in \left[\begin{array}{cc} 1 & 25 \end{array}\right]$ min, $\sigma_{m}\in \left[\begin{array}{cc} 0.5 & 1.5 \end{array}\right]$ u. Here, “u” means some arbitrary units, for example, deg/s or deg/h in the case of gyro errors, or m/s^2^ in the case of accelerometer errors. In the second case, the white-noise component is assumed to be known with RMS deviation $\sigma_{v}^{k}=0.5\;u.,\;k=1,2$. In the third case, the RMS of the white-noise component is unknown and uniformly distributed in the domain $\sigma_{v}^{1,2}\in \left[\begin{array}{cc} 0.25 & 1 \end{array}\right]\;u.$

### 4.1. Results of Model Structure Identification Probability

The real (Equation (15)) and calculated (Equation (18)) identification error probabilities are computed using the method of statistical trials for all the three cases described above. This implies that the problem is solved using the proposed algorithm for 500 simulated samples for each case. 

**Figure 1:**
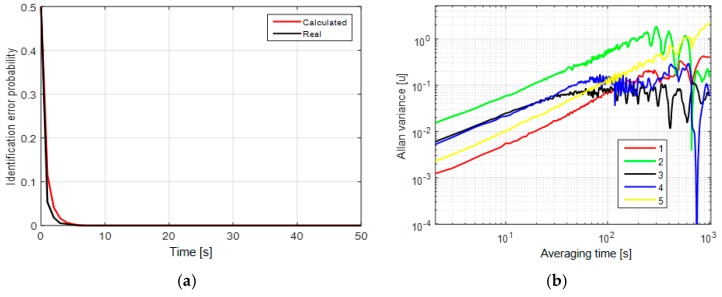
(**a**) Unconditional probability of the identification error without the white-noise component; (**b**) Allan variances for the random walk with $\sigma_{w}^{}=0.04$ u (1—red), $\sigma_{w}^{}=0.15$ u (2—green) and the first-order Markovian process with τ=1 min, $\sigma_{m}^{}=0.5$ u (3—black), τ=5 min, $\sigma_{m}^{}=1$ u (4—blue), τ=25 min, $\sigma_{m}^{}=1.5$ u (5—yellow).

In the first case, the mean probability of the identification error is close to zero after a couple of measurements (Figure 1a). Thus, these processes can be easily identified. To illustrate that not all methods allow us to do so, we give the AV [9,12] of these processes in Figure 1b. The AV [9,12] are obtained using a complete set of measurements of length 2000 s. The model identification using AV usually consists in empirical finding of significant slopes in the plot. We can hardly find difference between the AV for different models in Figure 1b because we can see only +1/2 slope in this plot, but there is no significant slope −1/2 of the first-order Markovian process. Taking into consideration the correlation interval of the first-order Markovian process, the slope −1/2 starts after the averaging time of 200 s, where AV is unreliable because of the sample length of 2000 s. To distinguish between these processes using AV, we should take a sample exceeding 20,000 s in length. 

**Figure 2:**
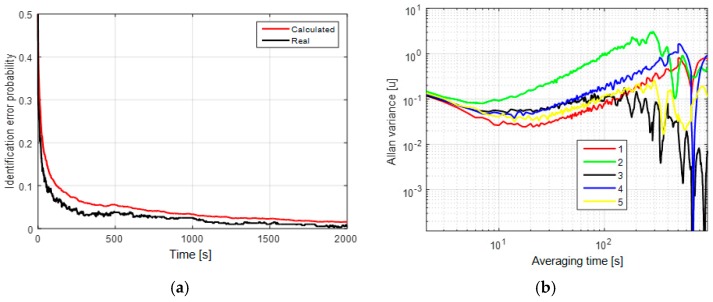
(**a**) Unconditional probability of the identification error with white-noise component of known RMS; (**b**) Allan variances for the sums of white noises with $\sigma_{v}^{}=0.5$ u and the random walk with $\sigma_{w}^{}=0.04$ u (1—red), $\sigma_{w}^{}=0.15$ u (2—blue) and with the first-order Markovian process with τ=1 min, $\sigma_{m}^{}=0.5$ u (3—black), τ=5 min, $\sigma_{m}^{}=1$ u (4—blue), and τ=25 min, $\sigma_{m}^{}=1.5$ u (5—yellow).

**Figure 3:**
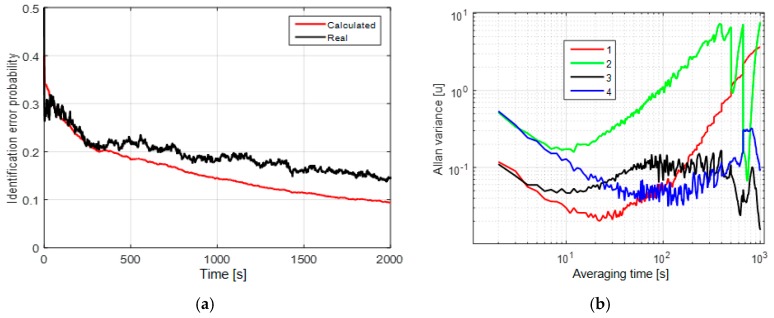
(**a**) Unconditional probability of the identification error with the white-noise component of RMS $\sigma_{v}^{1,2}\in \left[0.25\;1\right]$ u; (**b**) Allan variances for the sums of white noises and the random walk with $\sigma_{w}^{}=0.04$ u, $\sigma_{v}^{}=0.5$ u (1—red), $\sigma_{w}^{}=0.15$ u, $\sigma_{v}^{}=1$ u (2—green) and with the first-order Markovian process with τ=1 min, $\sigma_{m}^{}=0.5$ u, $\sigma_{v}^{}=0.5$ u (3—black), τ=25 min, $\sigma_{m}^{}=1.5$ u, $\sigma_{v}^{}=1$ u (4—blue).

In the second and third cases, the mean probabilities of the identification error after 2000 s of measurements are about 2% (Figure 2a) and 10% (Figure 3a), respectively. Here, we may conclude that the uncertainty of the white-noise component affects significantly the model identification probability for these two models. The AV for these cases have two significant slopes: −1/2 and +1/2 that correspond to the white noise and random walk (Figure 2b and Figure 3b).

Thus, we can separate white noise and correlated processes, but the random walk and the first-order Markovian processes can hardly be distinguished with the use of AV as in the first case. Note that for the second and third cases, we did not set apart the white noise as a separate hypothesis, which is why we did not calculate the probability of its detection directly. Hence, the possibility of white-noise component identification by using the proposed algorithm can be judged from the corresponding RMS error of the white-noise component, which is presented in the next subsection.

It is shown that the first-order Markovian process and random walk cannot be distinguished by AV for all three cases. These results show that the potential accuracy of model and parameter identification can be much higher than the accuracy provided with the use of AV. The criteria for the performance can be calculated as unconditional error probabilities (Equations (15) and (18)). Figure 1a, Figure 2a and Figure 3a also show the coincidence of the real and calculated identification probabilities, which implies that the calculated values are reliable for estimation of identification probability in a real-data experiment. 

### 4.2. Results of Parameter Estimation Accuracy

**Figure 4:**
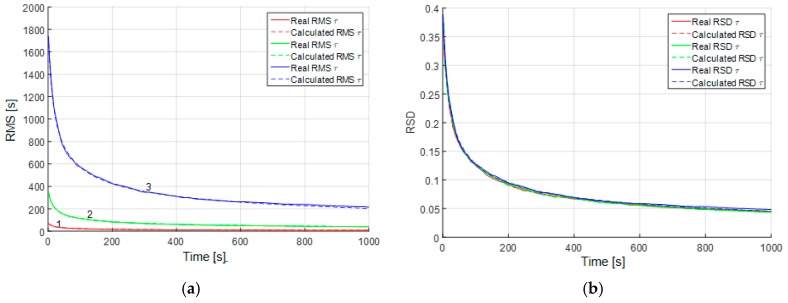
(**a**) Estimation error RMS for the correlation interval of the Markovian process with different initial uncertainties: 1 (red)—$\tau_{m}\in \left[\begin{array}{cc} 1 & 5 \end{array}\right]$ min, 2 (green)—$\tau_{m}\in \left[\begin{array}{cc} 5 & 25 \end{array}\right]$ min, 3 (blue)—$\tau_{m}\in \left[\begin{array}{cc} 25 & 125 \end{array}\right]$ min; (**b**) estimation error RSD for the correlation interval of the Markovian process with the same parameters.

The mean accuracy characteristics for parameter estimation can also be calculated using the proposed algorithm. This subsection is devoted to the study of accuracy in different conditions. However, before we proceed to the analysis of parameter estimation accuracy, let us show how the results of such analysis can be generalized using RSD. Consider Equations (22) and (23) with different uncertainty ranges of parameters. For example, let $\tau_{m}$ be uniformly distributed within the range of $\left[\begin{array}{cc} 1 & 5 \end{array}\right]$ min in the first case, $\tau_{m}\in \left[\begin{array}{cc} 5 & 25 \end{array}\right]$ min in the second case, and $\tau_{m}\in \left[\begin{array}{cc} 25 & 125 \end{array}\right]$ min in the third case. For these cases we can obtain calculated and real RMS (Figure 4a). 

It can be seen from the plots (Figure 4a) that the RMS of the estimation errors are substantially different due to different true values of the correlation interval and the level of initial uncertainty. However, the RSD (Figure 4b) for different sizes of the prior uncertainty domain of parameter $\tau_{m}$ coincide. Thus, the result obtained for a parameter with the initial uncertainty domain $\theta \in \left[\begin{array}{cc} \theta_{1} & \theta_{2} \end{array}\right]$ can be generalized for all domains of the form $\theta \in \left[\begin{array}{cc} r\theta_{1} & r\theta_{2} \end{array}\right]$, where r is an arbitrary real number different from zero. Thus, we use RSD as an accuracy characteristic in the following plots.

**Figure 5:**
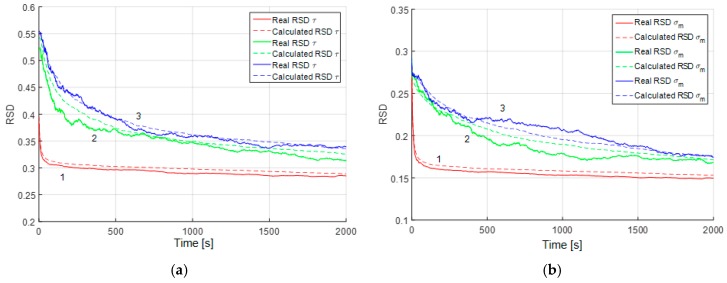
Real and calculated RSD for the parameters of the Markovian process: (**a**) Correlation interval; (**b**) RMS, 1 (red)—without white-noise component, 2 (green)—with white-noise component of known RMS, 3 (blue)—with white-noise component of unknown RMS (3).

**Figure 6:**
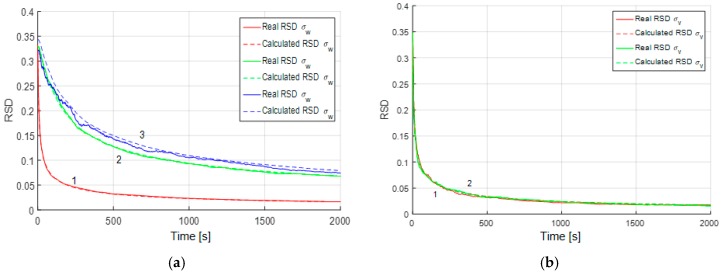
(**a**) Real and calculated RSD for RMS deviation of system noise for the random walk: 1 (red)—without white-noise component, 2 (green)—with white-noise component of known RMS, 3 (blue)—with white-noise component of unknown RMS; (**b**) Real (1—red) and calculated (1—red dashed) RSD for white-noise component RMS estimates with model (21) and Real (2—green) and calculated (2—green dashed) RSD for white-noise component RMS estimates with Equation (22).

Figure 5 and Figure 6 show the RSD for the estimates of parameters for the both models and all three cases considered above. It may be inferred that in the presence of white noise, the accuracy of the parameter estimation does not vary much regardless of whether the RMS noise is known or not. The greatest influence that causes decrease in the estimation accuracy is explained by the presence of the white-noise component. 

In the third case, the RMS of the white-noise component is estimated with a rather high accuracy, which is the same for the both alternative models (Figure 6b). It should be noted that if we try to estimate the RMS of the white noise in a sample without white noise, such as in the first simulation case, the white noise RMS estimates will converge to zero. The above facts allow us to conclude that the white-noise component is easy to detect both with the use of the proposed algorithm and AV. Note that in Figure 4, Figure 5 and Figure 6 the calculated and real RSD coincide, which is evidence of the validity of the results obtained and reliability of the calculated values.

## 5. Conclusions

The Bayesian approach to identification of sensor error models based on the solution of a joint hypotheses recognition and parameter estimation problem is considered. The following main advantages of this approach are emphasized: possibility of estimating an unknown structure and parameters of a model at any time interval both for stationary and nonstationary processes; capability of calculating characteristics for estimation accuracy; the possibility of obtaining a model in the form that is convenient for solution of the estimation problem using stochastic filtering algorithms.

In the framework of this approach, the paper proposes the performance criteria for the identification problem of sensor error models in the form of an unconditional probability for identification of a true model structure and a relative standard deviation (RSD) for unknown parameters of the model. The probability of choosing the correct structure of the model is defined as the probability of choosing a shaping filter corresponding to the correct model. The RSD of unknown parameters are determined using the unconditional covariance matrices calculated for the chosen filter. The introduced criteria create preconditions for an objective study of the performance of the identification algorithm depending on the initial data. This analysis should be carried out to evaluate the possibility of the identification problem solution under given conditions, including a given set of hypotheses, or to choose the conditions that provide a satisfactory solution. 

An example of the identification problem solution is given for the models that are traditionally used to describe errors of navigation sensors. The models are represented as a sum of correlated processes specified by the random walk or the first-order Markovian process, white-noise and constant components. The dependencies in the probability of correct model identification and the accuracy of parameter estimation from the RMS of the white-noise component are analyzed, including the case when the RMS is not precisely known and should be identified during the problem solution. It has been shown that the probability of model identification largely depends on the presence of the white-noise component. At the same time, the fact of uncertainty in the knowledge of the white-noise component RMS has an insignificant effect on the probability of model identification and the accuracy of parameter estimation.

In our future work the proposed criteria will be used to evaluate the performance of suboptimal algorithms, such as the Allan variance, for the problems of estimating error models of navigation sensors. This study will involve the comparison of suboptimal and the proposed algorithms.
